# Supplementary material for: Captive-rearing changes the gut microbiota of the bumblebee Bombus lantschouensis native to China
Source: PeerJ. 2025 Feb 13;13:e18964. doi: 10.7717/peerj.18964 (PMC11830364; doi:10.7717/peerj.18964)
Supplement: Supplemental Information 1 [file peerj-13-18964-s001.docx]

**Supporting Information**


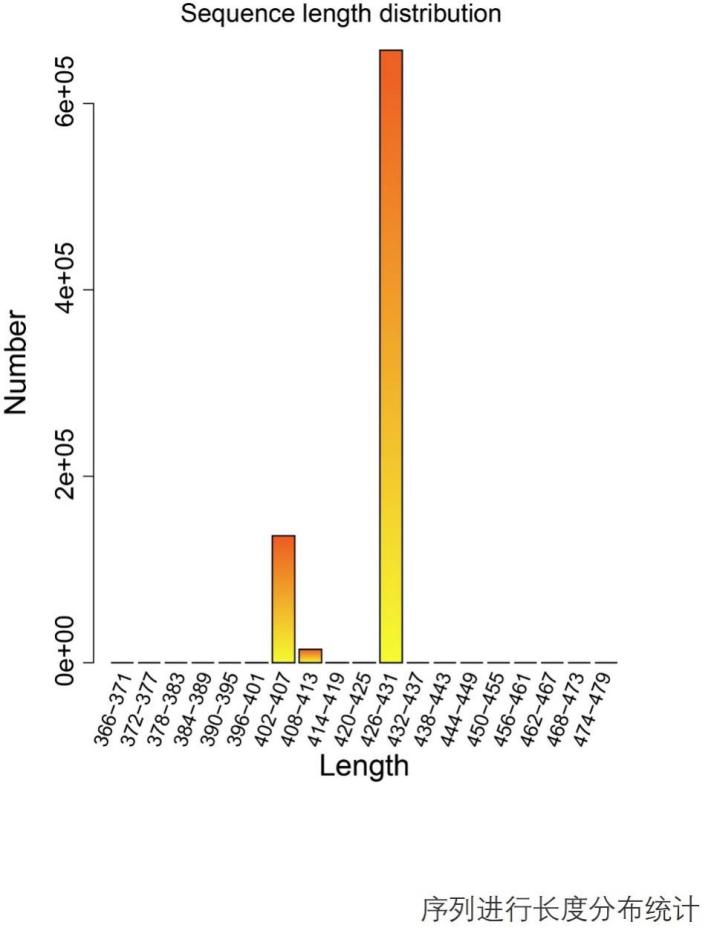


**Figure S1. The statistic of ASV sequences in the gut microbiota of *B. lantschouensis*.** The sequence length distribution of ASV sequences in the gut microbiota of *B. lantschouensis*.


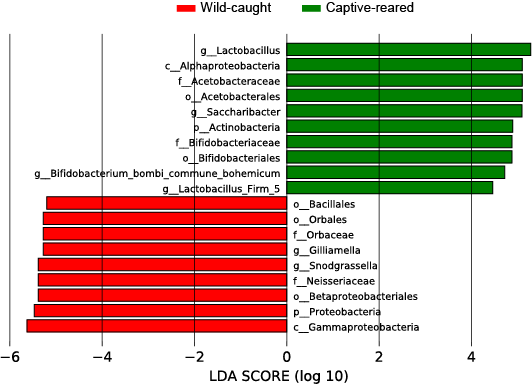


**Figure S2.** The differentially abundant bacteria among samples with different treatment groups. LDA scores (bacteria that obtain a log LDA score of >4 are ultimately considered) can be interpreted as the degree of consistent difference in relative abundance among two treatments.


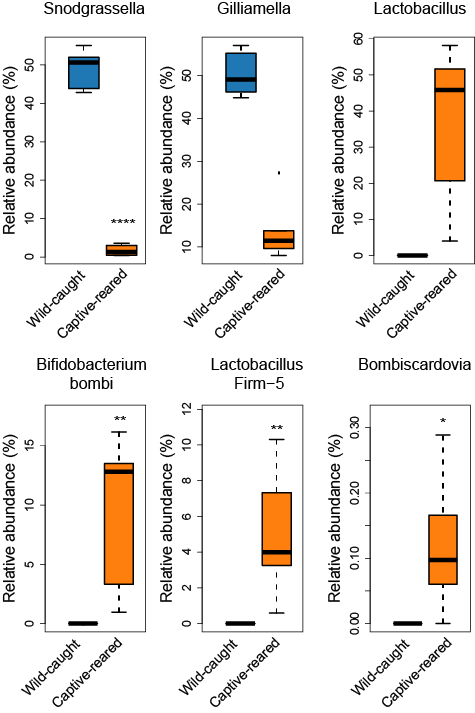


**Figure S3.** the most significant difference of genera in wild-caught group and captive-reared group of *B. lantschouensis* at the genus level*.*


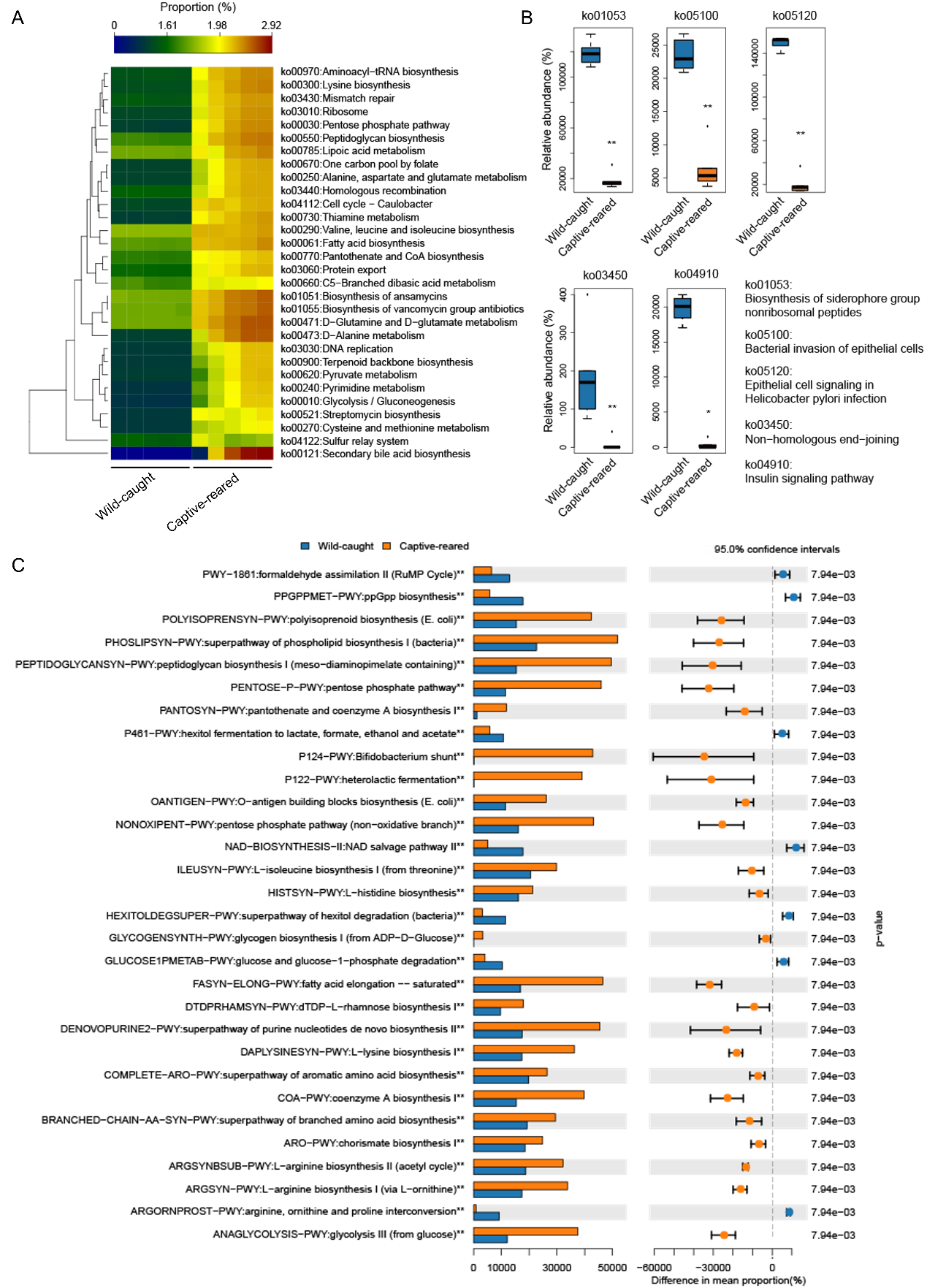


**Figure S4. Functional prediction of gut microbiota with** **Captive-Reared in *B. lantschouensis*. (A)** The Heatmap of KEGG function of gut microbiota in wild-caught group and captive-reared group of *B. lantschouensis*. **(B)** The histogram of functional subcategories showed a lower abundance after captive-reared of *B. lantschouensis*. **(C)** Histogram of functional subcategories with the top 30 metabolic pathways of gut microbiota by MetaCyc between two groups of *B. lantschouensis* (p<0.05). Significant differences in relative abundance of functions predicted between two groups.

**Table 1. Summary of quality control in 16S rRNA amplicon sequencing of gut microbiota in *B. lantschouensis*.**

| Id | Group | Input | Filtered | Percentage of input passed filter | Denoised | Merged | Percentage of input merged | Non-chimeric | Percentage of input non-chimeric |
| --- | --- | --- | --- | --- | --- | --- | --- | --- | --- |
| 1 | Wild-Caught | 83586 | 76803 | 91.89 | 76647 | 76352 | 91.35 | 69110 | 82.68 |
| 2 | Wild-Caught | 81330 | 74475 | 91.57 | 74387 | 74186 | 91.22 | 68051 | 83.67 |
| 3 | Wild-Caught | 84237 | 75050 | 89.09 | 74935 | 74706 | 88.69 | 69649 | 82.68 |
| 4 | Wild-Caught | 81290 | 74738 | 91.94 | 74619 | 74307 | 91.41 | 68170 | 83.86 |
| 5 | Wild-Caught | 77502 | 70531 | 91.01 | 70403 | 70099 | 90.45 | 66138 | 85.34 |
| 6 | Captive-Reared | 79229 | 72589 | 91.62 | 72459 | 71891 | 90.74 | 68114 | 85.97 |
| 7 | Captive-Reared | 74431 | 69147 | 92.9 | 69022 | 68883 | 92.55 | 66244 | 89 |
| 8 | Captive-Reared | 76128 | 70650 | 92.8 | 70494 | 70015 | 91.97 | 66681 | 87.59 |
| 9 | Captive-Reared | 76018 | 70852 | 93.2 | 70608 | 70007 | 92.09 | 66512 | 87.5 |
| 10 | Captive-Reared | 87345 | 75059 | 85.93 | 74963 | 74405 | 85.19 | 69420 | 79.48 |

**Table 2. The abundance of sequences annotated at the genera level in wild-caught group and captive-reared group of *B. lantschouensis.***

| Genus | Abundance Of Sequences | |
| --- | --- | --- |
|  | Captive-Reared | Wild-Caught |
| Gilliamella | 8627 | 31054 |
| Snodgrassella | 1091 | 30055 |
| Lactobacillus | 22180 | 16 |
| Saccharibacter | 17345 | 15 |
| Bifidobacterium bombi/commune/bohemicum | 5945 | 1 |
| Lactobacillus Firm-5 | 3313 | 0 |
| Unassigned | 2650 | 122 |
| Gluconobacter | 261 | 0 |
| Staphylococcus | 0 | 227 |
| Bombiscardovia | 79 | 0 |
| Carnimonas | 12 | 0 |
| Phaseolibacter | 0 | 9 |
| Acinetobacter | 0 | 4 |
| Lactobacillus kunkeei | 0 | 3 |
